# Evaluation of adaptation based on biological variability and substantial changes (SCC) in young rhythmic gymnasts

**DOI:** 10.3389/fspor.2026.1860505

**Published:** 2026-07-15

**Authors:** Kadirov Rashid Xamidovich, Abdullayev Mehriddin Junaydulloyevich, Xusenov Nodir Nuriddinovich, Djuraeva Makhasti Zokir kizi, Rustamov Akbar Askarovich, Moʻminov Feruzjon Ilxomovich

**Affiliations:** 1 Department of Sports Theory and Methodology, Bukhara State University, Bukhara, Uzbekistan; 2 Department of Theory and Methodology of Physical Culture, Bukhara State Pedagogical Institute, Bukhara, Uzbekistan

**Keywords:** biological age, Classical Test Theory (CTT), functional asymmetry, measurement reliability, minimal detectable change (MDC), ontogenetic asynchrony, postural control, rhythmic gymnastics

## Abstract

**Background:**

In rhythmic gymnastics, assessing physical fitness during the early specialization phase (ages 5–10 years) traditionally relies on chronological age and group averages. This approach frequently yields false-positive conclusions (Type I errors) due to ontogenetic asynchrony and pronounced biological variability. This study aims to transcend the limitations of traditional statistical models by establishing true motor adaptation thresholds in young gymnasts using the minimal detectable change (MDC) metric.

**Methodology:**

The study included 33 female athletes in a foundational rhythmic gymnastics training group. Initially, anthropometric screening was utilized to quantify discrepancies between chronological and biological age. The reliability of both physical and postural tests was evaluated using Classical Test Theory (CTT) metrics, employing a test-retest design with a 7-day interval. To account for functional body asymmetry, data were analyzed based on dominant and non-dominant sides rather than anatomical left or right distinctions, calculating the standard error of measurement (SEM) and MDC.

**Results:**

Anthropometric analysis confirmed the presence of significant somatic asynchrony—with 24% of the cohort exhibiting accelerated maturation and 12% showing delayed maturation (retardation)-leading to stochastic variance in baseline performance parameters. Passive flexibility tests demonstrated high measurement stability (ICC = 0.90). In static balance assessments, reliability differed significantly due to neural lability; the dominant leg (ICC = 0.78, MDC = 18.0 s) showed markedly higher stability compared to the non-dominant leg (ICC = 0.65, MDC = 21.8 s). Furthermore, the MDC threshold for explosive power in the standing long jump was established at 11.9 cm.

**Conclusion:**

Relying solely on chronological age and traditional significance testing (*p*-values) risks misinterpreting random developmental variance as a training effect in youth sports. Implementing individualized MDC thresholds, appropriately adjusted for neuromotor asymmetry, provides coaches with a highly reliable tool to objectively assess true training adaptations and prevent excessive physical strain.

## Introduction

Modern sports metrology is undergoing a fundamental paradigm shift toward highly individualized assessments of training efficacy. Accurately evaluating the physical fitness of young athletes is particularly critical in early-specialization sports that require complex coordination, such as rhythmic gymnastics. This unique sport demands exceptional passive and active flexibility, lower-limb explosive power, and impeccable postural control (1–3).

The developmental period between 5 and 10 years of age represents a crucial neurophysiological window for the rapid maturation of the neuromuscular system and the establishment of foundational motor control patterns (4–6). During this phase, the development of static and dynamic balance, alongside motor coordination, provides the scaffolding necessary for mastering highly complex acrobatic and choreographic elements in later stages (7, 8). However, conventional fitness assessment methods in sports pedagogy predominantly rely on group arithmetic means, failing to account for the substantial intra-individual biological variability inherent in school-aged children.

In youth sports, chronological age is frequently an unreliable indicator of true somatic and biological maturity (9). The ontogenetic asynchronization in the maturation rates of skeletal and musculofascial tissues generates significant stochastic fluctuations, or “biological noise,” in physical testing outcomes (10, 11). Furthermore, due to the ongoing myelination of central nervous system pathways governing multisensory integration, a young gymnast's ability to maintain balance is heavily influenced not only by physical endurance but also by cognitive focus, neural lability, and functional asymmetry (lateralization).

For decades, statistical analysis in sports science has relied heavily on null hypothesis significance testing (NHST) and traditional *p*-values <0.05. However, in small cohorts characterized by high biological noise, standard parametric tools such as Student's *t*-tests or ANOVA exhibit severe methodological limitations. Relying exclusively on *p*-values often obscures the true magnitude of a training effect and individual adaptive responses. Consequently, researchers have explored alternative models like magnitude-based inference (MBI) to emphasize practical relevance (12, 13); however, rigorous systematic analyses have demonstrated that MBI unacceptably inflates the risk of Type I errors, leading to false-positive conclusions (14, 15).

A methodologically robust solution to this analytical crisis lies in Classical Test Theory (CTT), specifically through the application of relative and absolute reliability models (16, 17). Recent literature highlights the appropriateness of utilizing the standard error of measurement (SEM) and minimal detectable change (MDC) metrics when evaluating physical capabilities in children and adolescents (18–21). The MDC serves as a rigorous mathematical filter: any performance variation that does not exceed this specific threshold is rejected as stochastic biological fluctuation or instrumental error, rather than true physiological adaptation.

To practically address this methodological gap, the present study employs a systematic three-step analytical framework. First, anthropometric screening is utilized to differentiate chronological from biological age, effectively quantifying ontogenetic asynchrony. Second, the study evaluates how this biological variance manifests as statistical dispersion in physical and postural tests, particularly concerning lateralization between dominant and non-dominant sides. Finally, by calculating the intraclass correlation coefficient (ICC) and MDC, the study establishes individual internal stability and actionable growth thresholds. The ultimate aim of this research is to evaluate the reliability of baseline motor and postural tests in a cohort of young rhythmic gymnasts (*n* = 33) and to model their true adaptation thresholds using MDC, accounting for somatic asynchrony. This approach advocates for a paradigm shift from traditional *p*-value dependence to individualized MDC analysis, thereby radically improving the precision of pedagogical monitoring and training management.

The aim of this study was to evaluate the reliability of baseline motor performance and posture tests based on the aforementioned three-stage architecture in a larger sample (*n* = 33) of young rhythmic gymnasts at the initial stage of training and to model their actual minimally observable change (MDC) adaptation thresholds, taking into account somatic asynchrony. This study aims to demonstrate that switching from the traditional *p*-value paradigm to individual MDC analysis radically improves the quality of testing and training management for young athletes.

## Methodology

### Study design and participants

This study employed a repeated-measures (test-retest) design to evaluate the intra-individual reliability of physical and postural tests, aiming to establish true motor adaptation thresholds. The sample consisted of 33 young female athletes engaged in foundational rhythmic gymnastics training. Prior to data collection, written informed consent was obtained from the athletes' parents or legal guardians, and the study was conducted in accordance with the Declaration of Helsinki. In this specific age cohort, relying on traditional between-group comparisons (e.g., *t*-tests or ANOVA) is methodologically flawed, as significant intra-individual biological variability often obscures true training effects within statistical “noise” (14). Therefore, the study design was anchored in Classical Test Theory (CTT) to rigorously account for within-subject variance.

### Chronological and biological age determination

As a primary analytical step, a quantitative assessment of the athletes' somatic asynchrony was conducted. First, the exact chronological age (CA) was calculated in decimal format for each gymnast by subtracting the date of birth $\left(D_{birth}\right)$ from the date of testing $\left(D_{test}\right)$ and dividing by the international constant of 365.25 to account for leap years (9):

$$CA=\frac{D_{test}\;-\;D_{birth}}{365.25}$$

Secondly, to eliminate the radiation risks associated with skeletal x-rays, biological age (BA) was determined using a validated non-invasive anthropometric method (10). The absolute height and body mass of the gymnasts were evaluated against the 50th percentile of the World Health Organization's growth standards for girls (11). Finally, by calculating the discrepancy between biological and chronological age (Δ=BA−CA), the athletes were classified into three somatic development categories: normal maturation (−0.5≤Δ≤0.5years), accelerated maturation (Δ≥0.6years), and delayed maturation or retardation (Δ≤−0.6years).

### Standardization of physical and postural tests

The physical readiness of the young gymnasts was assessed using four specialized motor tests (1):

Longitudinal Split Test (cm): Evaluates the passive mobility and flexibility of the pelvic joints.

“Bridge” Test (cm): Measures spinal extension in the sagittal plane and shoulder girdle mobility.

Flamingo Static Balance Test (seconds): Assesses multisensory integration and core postural control.

Standing Long Jump Test (cm): Quantifies the explosive power of the lower extremities.

To ensure the accurate verification of reliability indices, the test battery was repeated with a strict 7-day interval (Test 1: December 15, 2025; Test 2: December 22, 2025) (17). Testing conditions were rigorously standardized to minimize exogenous variables that could artificially inflate the standard error of measurement. All assessments were conducted within the same diurnal window (15:00–17:00) to control for circadian rhythm fluctuations. Furthermore, aggressive passive stretching was deliberately excluded from the 15-minute standardized warm-up to prevent stretch-induced strength deficits during the explosive jump tests.

### Lateralization and asymmetry control protocol

In rhythmic gymnastics, functional neuromotor asymmetry heavily influences performance outcomes. To reduce statistical variance and prevent the artificial deflation of intraclass correlation coefficients (ICC), the unilateral tests (Longitudinal Split and Static Balance) were analyzed based on functional dominance (dominant vs. non-dominant sides) rather than anatomical left or right distinctions (18). The side demonstrating superior and more stable performance was categorized as dominant. Consequently, all statistical metrics (Mean, SD, SEM, and MDC) were calculated independently for the dominant and non-dominant sides. This methodology is critical for establishing side-specific adaptation thresholds, given that the non-dominant side inherently exhibits greater neural lability and measurement error.

### Statistical analysis and MDC modeling

To distinguish genuine motor adaptation from random biological maturation, the test-retest data were analyzed using CTT metrics. Initially, the relative reliability was determined using the Intraclass Correlation Coefficient (ICC). A two-way mixed-effects model based on absolute agreement $\left(ICC_{3.1}\right)$ was applied, as it properly accounts for ontogenetic age differences as natural systemic variance rather than mere error (16).

Subsequently, absolute reliability—expressed in the physical units of the respective tests—was calculated as the Standard Error of Measurement (SEM) using the pooled standard deviation $\left(SD_{\;pooled}\right)$ (19):

$$SEM=SD_{\;pooled}\;x\;\sqrt{1\;-\;ICC}$$

Ultimately, to guide practical pedagogical decision-making, the Minimal Detectable Change at a 95% confidence level $\left(MDC_{95}\right)$ was modeled. This metric utilizes a *z*-score of 1.96 and the constant $\sqrt{2}$ to account for the variance across the two repeated measurements (19):

$$MDC_{95}=SEM\;x\;1.96\;x\;\sqrt{2}$$

The $MDC_{95}$ effectively filters out general biological variance, establishing a strict individual growth threshold. Any performance improvement must mathematically exceed this threshold to be objectively interpreted as a true pedagogical adaptation resulting from training, rather than stochastic developmental noise. Statistical analyses were performed using IBM SPSS Statistics (Version 27.0) and Microsoft Excel.

## Results

Chronological vs. Biological Age and Ontogenetic Asynchrony At the initial stage of the study, the discrepancy between chronological (CA) and biological age (BA) was analyzed in a cohort of 33 young gymnasts. The results demonstrated a pronounced somatic asynchrony within the group. Although the mean chronological age of the athletes was 7.0 ± 1.2 years, their biological age was distributed across an extremely wide range, spanning from 5.1 to 11.1 years. The observation that approximately 24% of the sample exhibited accelerated maturation (Δ>+0.6), while 12% showed delayed maturation or reardation (Δ<−0.6), provides clear empirical evidence that the neuromuscular systems and somatic maturity of the girls are at markedly different stages of development. The chronological and biological age characteristics of the participants are presented in Table 1.

**Table 1:** Indicators of chronological and biological age of gymnasts (*n* = 33).

No.	Date of birth	Chronological age (CA)	Height (cm)	Weight (kg)	Biological age (BA)	Difference (Δ)	Developmental feature
1	June 17, 2016	9.5	136	29.5	9.8	+0.3	Normal
2	Sept 10, 2018	7.3	127	25.0	8.1	+0.8	Acceleration
3	Oct 9, 2019	6.2	115	20.0	6.1	−0.1	Normal
4	June 18, 2018	7.5	121	22.0	7.1	−0.4	Normal
5	August 1, 2018	7.4	132	27.0	8.4	+1.0	Acceleration
6	Sept 30, 2018	7.2	123	23.5	7.4	+0.2	Normal
7	August 2, 2018	7.4	126	25.0	7.9	+0.5	Normal
8	April 11, 2017	8.7	132	26.5	9.1	+0.4	Normal
9	June 10, 2019	6.5	124	24.5	7.4	+0.9	Acceleration
10	Sept 29, 2016	9.2	130	25.0	8.4	−0.8	Retardation
11	Sept 26, 2019	6.2	111	18.5	5.6	−0.6	Retardation
12	July 18, 2020	5.4	109	17.0	5.1	−0.3	Normal
13	August 29, 2016	9.3	133	27.0	9.1	−0.2	Normal
14	Dec 4, 2019	6.0	113	19.5	5.8	−0.2	Normal
15	August 1, 2020	5.4	112	18.5	5.6	+0.2	Normal
16	Oct 7, 2019	6.2	120	22.5	6.8	+0.6	Acceleration
17	August 2, 2019	6.4	117	20.5	6.2	−0.2	Normal
18	Feb 11, 2020	5.8	110	18.0	5.4	−0.4	Normal
19	May 12, 2018	7.6	127	24.5	8.1	+0.5	Normal
20	Nov 23, 2019	6.1	110	17.5	5.4	−0.7	Retardation
21	Feb 5, 2017	8.9	130	25.5	8.5	−0.4	Normal
22	August 14, 2018	7.3	134	28.0	8.5	+1.2	Acceleration
23	Jan 30, 2020	5.9	117	21.0	6.4	+0.5	Normal
24	April 19, 2018	7.7	124	23.5	7.5	−0.2	Normal
25	July 8, 2019	6.4	121	22.0	6.9	+0.5	Normal
26	Sept 25, 2017	8.2	135	29.0	9.1	+0.9	Acceleration
27	Dec 3, 2018	7.0	119	21.5	6.7	−0.3	Normal
28	March 16, 2019	6.8	118	20.0	6.5	−0.3	Normal
29	June 21, 2018	7.5	123	23.0	7.3	−0.2	Normal
30	Oct 11, 2019	6.2	116	19.5	6.0	−0.2	Normal
31	Jan 2, 2017	9.0	127	24.0	7.9	−1.1	Retardation
32	May 28, 2020	5.5	114	19.0	5.9	+0.4	Normal
33	August 17, 2018	7.3	126	24.0	7.6	+0.3	Normal

Stochastic Variance in Physical and Postural Tests This within-group ontogenetic asynchrony directly determined significant statistical dispersion in the outcomes of both motor and postural tests, as shown in Table 2. According to established statistical principles, a high standard deviation (SD) indicates the presence of substantial “biological noise” within the sample.

**Table 2:** Descriptive statistics of physical and postural tests (test and retest).

Test name	Lateralization	Test 1 (mean ± SD)	Test 2 (mean ± SD)
Longitudinal split (cm)	Dominant	13.5 ± 5.2	13.1 ± 4.9
	Non-dominant	8.2 ± 4.1	7.9 ± 3.8
Bridge (cm)	Bilateral	13.2 ± 5.8	13.5 ± 5.4
Static equilibrium (s)	Dominant	32.4 ± 14.1	30.8 ± 13.5
	Non-dominant	18.6 ± 12.3	19.2 ± 14.0
Standing long jump (cm)	Bilateral	104.3 ± 12.8	105.1 ± 11.9
Arm flexion and extension	Bilateral	14.8 ± 4.7	15.2 ± 4.5
Jumping rope (20 s)	Bilateral	39.5 ± 3.6	40.1 ± 3.4

Baseline measurements demonstrated that standing long jump results, an indicator of explosive lower-limb power, ranged widely from 83 to 123 cm, producing a high standard deviation of ±12.8 cm. The most pronounced stochastic instability was observed in the static balance test, where individual performances fluctuated drastically across a polar range of 0–50 s. With such extreme standard deviations, it is empirically evident that traditional hypothesis-testing models based on group means (e.g., *p* < 0.05) lose their statistical power, leading to a high probability of false-positive (Type I) errors when attempting to verify true training efficacy.

Modeling the Adaptation Threshold Using CTT and MDC Metrics. To methodologically overcome these limitations, the test-retest data (collected at a 7-day interval) were processed using the framework of Classical Test Theory (CTT). To rigorously filter out random biological variations and instrumental measurement errors, absolute reliability parameters were calculated, specifically, the standard error of measurement (SEM) and the minimal detectable change (MDC) thresholds at a 95% confidence interval for each test, taking into account functional body asymmetry. The relative and absolute reliability estimates are presented in Table 3.

**Table 3:** Relative and absolute estimates of reliability for diagnostic tests (*n* = 33).

Test name	Lateralization	Relative reliability: ICC (95% CI)	Absolute reliability: SEM	Adaptation threshold: MDC (95%)
Longitudinal split (cm)	Dominant	0.92 (0.85–0.96)	1.4 cm	3.8 cm
	Non-dominant	0.87 (0.78–0.93)	1.8 cm	5.0 cm
Bridge (cm)	Bilateral	0.94 (0.88–0.97)	1.3 cm	3.6 cm
Static equilibrium (s)	Dominant	0.78 (0.61–0.88)	6.5 s	18.0 s
	Non-dominant	0.65 (0.41–0.80)	7.9 s	21.8 s
Standing long jump (cm)	Bilateral	0.88 (0.76–0.94)	4.3 cm	11.9 cm
Arm flexion and extension	Bilateral	0.85 (0.71–0.92)	1.8 reps	5.0 reps
Jumping rope (20 s)	Bilateral	0.81 (0.65–0.90)	1.5 reps	4.1 reps

The analysis indicated that test reliability varied significantly depending on the physiological specificity of the task. The Bridge and Longitudinal Split tests (dominant side), which assess the elasticity of passive tissues, demonstrated the highest rating stability (ICC > 0.90) and minimal measurement error (SEM < 1.5 cm). For the standing long jump test, which evaluates explosive strength, the MDC threshold was established at 11.9 cm. Consequently, a young gymnast's jump performance must improve by a minimum of 12 cm for the change to be confidently interpreted as genuine muscular adaptation rather than stochastic noise.

The most critical findings were revealed through the asymmetry control protocol. In the Static Balance test, the reliability of the dominant leg remained at an acceptable level (ICC = 0.78). In contrast, the reliability for the non-dominant leg dropped sharply (ICC = 0.65), correlating with an increased measurement error. Mathematically, confirming a genuine improvement in postural balance on the dominant leg requires a minimum performance increase of 18.0 s; conversely, to confirm individual adaptation on the non-dominant leg, the athlete must improve by at least 21.8 s. This definitely proves that neuromotor asymmetry is highly pronounced in girls aged 6–8 years, and implementing individual MDC thresholds is the only way to make safe and objective pedagogical decisions when monitoring training dynamics.

Practical Application for Coaches: These empirical results underscore the urgent need to modify traditional pedagogical evaluation methods. Table 4 presents integrated hypothetical scenarios demonstrating how a practicing coach can utilize specific MDC thresholds to accurately distinguish random stochastic fluctuations from genuine neuromotor adaptations amidst the “biological noise” of young gymnasts.

**Table 4:** Practical application of MDC thresholds for monitoring fitness and lateral asymmetry.

Test conducted (side)	Initial test	Follow-up test (1 month)	Result increase (Δ)	Required MDC threshold	Objective pedagogical conclusion for the coach
Static balance (dominant leg)	20 s	35 s	+15 s	18.0 s	False positive. The improvement is attributed to daily neural lability (e.g., increased cognitive focus on the test day). A true objective training effect on posture control cannot be confirmed.
Static balance (non-dominant leg)	10 s	35 s	+25 s	21.8 s	True adaptation. The significant increase confirms a genuine improvement in neuromuscular coordination and an objective reduction in lateral asymmetry.
Standing long jump (explosive power)	100 cm	108 cm	+8 cm	11.9 cm	False positive. The 8 cm increase reflects normal physiological oscillation. A genuine training effect on explosive strength is not confirmed.
Longitudinal split (dominant leg)	10 cm	15 cm	+5 cm	3.8 cm	True adaptation. The extensibility of passive tissues has objectively increased, confirming the efficacy of the applied stretching program.
Arm flexion and extension (strength)	10 reps	14 reps	+4 reps	5.0 reps	False positive. An increase of 4 repetitions falls within the margins of group noise. A minimum improvement of 5 repetitions is required to confirm actual strength growth.

This conceptual model clearly illustrates the severe limitations of the traditional statistical approach (*p* < 0.05) in practical coaching scenarios. Typically, if a coach observes an 8 cm improvement in the long jump or a 15-second improvement in unilateral balance, they might mistakenly interpret these metrics as definitive evidence of a successful training program. However, mathematical modeling via MDC proves that girls aged 5–10 years possess such high neural lability that these specific shifts often represent mere stochastic noise. It is scientifically established that a coach must verify performance increases against these strict MDC thresholds to prevent confirmation bias and capture the true training effect. Implementing this rigorous algorithm protects children from physical overloading during early specialization and provides an objective, data-driven framework for monitoring the educational process.

## Discussion

From the perspective of classical statistical power analysis, a relatively small sample size of *n* = 33 is often considered insufficient to reliably detect significant test-retest variations using traditional significance testing (*p* < 0.05), significantly increasing the risk of statistical errors. However, instead of remaining bound to conventional *p*-value diagnostics, transitioning to advanced statistical markers—specifically the Standard Error of Measurement (SEM) and Minimal Detectable Change (MDC)—provides a far more effective and methodologically precise solution. By deploying the analytical logic of SEM and MDC, this study successfully circumvents the limitations of small sample sizes. It allows us to comprehensively and reliably prove that the observed performance shifts in the 33 athletes are genuine pedagogical training effects, rather than random stochastic noise.

The primary objective of this study was to methodologically overcome the limitations of traditional statistical models (NHST, based on *p*-values) when evaluating young gymnasts aged 5–10 years, and to model true adaptation thresholds using the Minimal Detectable Change (MDC) metric amidst ontogenetic asynchrony. The results demonstrate the critical need for a radical paradigm shift in pediatric sports metrology, providing a scientifically rigorous justification for transitioning toward an individual-centered analytical approach in sports pedagogy.

The most important aspect revealed in this study is that the significant statistical dispersion (variability) recorded in the motor and postural tests is not merely a localized phenomenon characteristic of this specific sample (*n* = 33), but rather reflects a fundamental biological pattern of ontogenesis. The development of a child's body is strictly governed by the law of ontogenetic heterochrony—the asynchronous maturation rate of various physiological systems and organs (9). It is precisely due to this “biological noise,” as emphasized by methodological critiques in sports science (14, 15), that conclusions based solely on group averages frequently lead to high rates of Type I errors (false-positive conclusions) in practical training scenarios.

The $MDC_{95}$ metric, modeled within the framework of Classical Test Theory (CTT), provides a mathematical mechanism to effectively “filter out” this biological noise. For instance, while the specific MDC threshold identified for the standing long jump (11.9 cm) is tailored to this particular cohort, the methodological imperative of utilizing MDC represents a universal evaluative standard. This ensures the fair and objective assessment of both accelerated and delayed-maturing athletes.

### Asymmetry: neurophysiological causes and motor control

The analysis of lateralization (asymmetry) during the postural control test yields conceptually significant implications for modern pediatric sports biomechanics. Consistent with previous findings on neuromotor asymmetry (4, 18), our empirical results demonstrated that the dominant and non-dominant limbs exhibit fundamentally different reliability metrics during balance maintenance. From a physiological perspective, this discrepancy does not merely reflect a localized “muscular weakness” on one side, but is primarily a manifestation of central nervous system (CNS) development.

Because the myelination of the central nervous system pathways in girls aged 5–10 years is not yet fully complete, the transmission of efferent and afferent signals from the cerebral cortex to the non-dominant leg is inherently less stable and slower compared to the dominant leg (7). It is this pronounced neural lability that directly inflates the random standard error of measurement (SEM) on the non-dominant side. Consequently, it was mathematically proven that while confirming a genuine balance improvement on the dominant leg requires a minimum increase of 18.0 s, true adaptation on the non-dominant leg requires a threshold of at least 21.8 s. This neurophysiological pattern strongly warns coaches against the gross methodological error of simply dividing or averaging bilateral performance metrics.

### Scientific and pedagogical significance for practice

Through its methodological solutions, this study serves as an evidence-based framework for practicing coaches. Under a traditional pedagogical approach, a coach observing a minor performance increase (e.g., +10 s in a unilateral balance test) might prematurely conclude that a training intervention was highly effective. However, the proposed MDC model objectively demonstrates that if this increment falls below the specific side's threshold, it does not represent genuine neuromotor adaptation. Instead, it likely reflects transient factors such as the child's daily cognitive focus, behavioral mood, or simple task-learning (habituation) to the testing procedure. During the critical early specialization stage in rhythmic gymnastics, MDC criteria act as the safest and most equitable pedagogical tool. They prevent the physical overloading of young athletes, ensure the targeted development of functional symmetry through compensatory exercises, and accurately differentiate between genuine muscular adaptation and temporary physiological fluctuation.

### Limitations and future directions

A primary limitation of this study is that the cohort (*n* = 33) consisted of athletes from a single sports specialization (rhythmic gymnastics) and a specific geographic location. Furthermore, the test-retest design, conducted with a 7-day interval, captures only the short-term relative and absolute reliability of the measurements. Future research should investigate the longitudinal stability of these modeled MDC thresholds over extended macrocycles, such as 6-month or 1-year training periods. Nevertheless, this study establishes a robust methodological foundation for pediatric sports metrology, successfully advocating for the necessary transition from traditional group-based statistical evaluations to a highly precise, individual-centered paradigm.

## Conclusion

Assessing the motor and postural potential of girls aged 5–10 years at the initial specialization stage in rhythmic gymnastics solely based on their chronological age constitutes a fundamental methodological error. The ontogenetic heterochrony and pronounced somatic asynchrony within the cohort—specifically, the varied states of accelerated and delayed maturation—demonstrate that any pedagogical program failing to account for these biological characteristics will inevitably lead to false-positive conclusions. Consequently, training and evaluation in youth sports must be grounded in biological age evaluations, determined via non-invasive anthropometric screening.

In the presence of the pronounced “biological noise” and stochastic dispersion typical of young athletes, traditional between-group comparisons relying on standard significance testing (*p* < 0.05) completely lose their statistical validity. The only objective mechanism to isolate a genuine training effect from random biological fluctuations and instrumental errors is the application of the Minimal Detectable Change $\left(MDC_{95}\right)$ algorithm. This metric, derived from Classical Test Theory (CTT), accurately reflects the true within-subject stability of performance.

Furthermore, this study established that in postural and unilateral motor tests, functional body asymmetry results in fundamentally different reliability metrics and independent adaptation thresholds for the dominant and non-dominant sides. The increased measurement error on the non-dominant side, driven by pronounced neural lability, necessitates a mathematically higher $MDC_{95}$ threshold (e.g., 21.8 vs. 18.0 s in the static balance test). This objectively confirms the physiological necessity of incorporating targeted compensatory exercises to mitigate lateral asymmetry during the early stages of training.

Ultimately, the individualized MDC criteria modeled in this research provide practicing coaches with a robust, scientifically validated methodological tool. Implementing these thresholds safeguards young female athletes from excessive physical loads during the critical early specialization phase, while ensuring the objective assessment of true neuromotor adaptation.

## Data Availability

The original contributions presented in the study are included in the article/Supplementary Material, further inquiries can be directed to the corresponding author.
